# Prevalence of dementia in a level 4 university epilepsy center: how big is the problem?

**DOI:** 10.3389/fneur.2023.1217594

**Published:** 2023-10-20

**Authors:** Christoph Helmstaedter, Teresa Lutz, Vinzent Wolf, Juri-Alexander Witt

**Affiliations:** Department of Epileptology, University Hospital Bonn (UKB), Bonn, Germany

**Keywords:** epileptology, epilepsy, seizures, dementia, cognition, neurodegeneration, Alzheimer's disease

## Abstract

**Background:**

The relationship between epilepsy and dementia is currently a topic of great interest. Our study aimed to determine the prevalence of dementia diagnoses among patients of a large level 4 university epilepsy center.

**Methods:**

In this retrospective monocentric study conducted at the Department of Epileptology of the University Hospital Bonn, we searched for dementia-related terms in a total of 145,501 medical letters from 40,360 adult patients who were seen between 2003 and 2021. Files with at least one hit were selected and analyzed with regard to diagnoses, age, age at epilepsy onset, and the question as to whether epilepsy preceded or followed the dementia diagnosis.

**Results:**

Among the medical letters of 513 patients, dementia-related terms were found. The letters of 12.7% of these patients stated a dementia diagnosis, 6.6% were suspected of having dementia, 4.9% had mild cognitive impairment, and 6.6% had other neurodegenerative diseases without dementia. Taking all 40,360 patients into account, the prevalence of diagnosed or suspected dementia was 0.25%. An older age (≥60 years) and late-onset epilepsy (≥60 years), but not a longer epilepsy duration, increased the odds of dementia by 6.1 (CI 3.5–10.7) and 2.9 (CI 1.7–4.7), respectively. Additionally, vascular, metabolic, inflammatory, and behavioral mood-related comorbidities were commonly observed. Epilepsy tended to precede (23.2%) rather than follow (8.1%) the dementia diagnosis.

**Conclusion:**

Despite the clear limitations of a selection bias and the potential underdiagnosis of dementia and underestimation of its prevalence when relying on the medical letters from a specialized center which rather focuses on epilepsy-related issues, the findings of this study offer valuable insights from the perspective of an epilepsy center. In this setting, the prevalence of dementia in epilepsy is rather low. However, physicians should be aware that the risk of dementia is higher in the elderly, in late-onset epilepsies, and when comorbid risk factors exist. Seizures can also be an early sign of a neurodegenerative disease. Future research should explicitly screen for dementia in patients with epilepsy and stratify them according to their underlying pathologies and comorbidities.

## Introduction

Cognitive impairment is a common comorbidity in epilepsy. In the majority of patients with any type of epilepsy, deficits can be observed in at least one cognitive domain (1). However, the relationship between epilepsy (i.e., having seizures) and cognition is complex and is bidirectional rather than a simple one-way relationship (2). Many factors can compromise neuropsychological function in epilepsy (3). In early-onset epilepsies, developmental hindrance is a major risk factor for global intellectual impairment (4). A long-standing discussion has been taking place as to whether chronic epilepsy and seizures have the potential to inflict brain damage, leading to a progressive and accelerated mental decline (5, 6). As early as the beginning of the twenteeth century, reports by Kraepelin (7) indicated that “epileptic dementia” was widespread and could affect up to 50% of patients with epilepsy. It was suggested that the numbers were even higher in what was referred to as “pure” or “real” epilepsy, which excluded symptomatic or structural epilepsy (8). While the numbers may seem very high, it is unclear what exactly led to the diagnosis of epileptic dementia at that time. Today, with an aging population, the issue of epileptic dementia is gaining renewed attention. This finds its expression in recent activities of the ILAE task force on epilepsy in the elderly which focus on epidemiology and epilepsy management (9, 10).

However, it is essential to differentiate between various possibilities such as whether dementing conditions cause epilepsy, whether seizures cause dementia, whether epilepsy and associated lesions lower the threshold for dementia, or whether epilepsy and dementia are comorbid conditions that share a common underlying factor (11). In some patients, factors related to seizures and their treatment can play a significant role in regard to mental decline (12–15). In other patients, indicators for neurodegeneration are found that can cause mental decline (16–18). A systematic review and meta-analysis of epidemiologic studies (19) among others (20, 21) on the relationship between epilepsy and dementia reported a period prevalence of epilepsy in dementia for 5 out of 100 persons compared to 4 out of 100 persons, population-based. Risk factors for developing epilepsy included severity, duration, and age of dementia onset. Of interest for the perspective taken here is the period prevalence of dementia in persons with epilepsy which ranges from 8.1 to 17.5, without exploring any risk factors. A recent long-term follow-up study [Atherosclerosis Risk in Communities (ARIC) Study] has provided evidence that the late onset of epilepsy (LOE, ≥ age 67) is a risk factor for future dementia (22, 23). In the long run, 42% of 671 individuals with LOE developed dementia. The hazard ratio for developing dementia was approximately three times higher for subjects with LOE vs. those without LOE. Evidence of an approximately 2-fold risk for developing epilepsy from dementia and vice versa resulted from an analysis of data from the Framingham Heart study, which included 4,905 patients with information on epilepsy and dementia, and dementia follow-up after the age of 65 years (24). A retrospective cohort study involving 675 individuals with epilepsy (aged ≥ 50 years) observed a 3-fold risk for developing dementia (25). Ophir et al. reported a 10-year cumulative incidence of dementia of 22% in patients with LOE of unknown etiology (26).

In this study, we shed light on this issue from an epileptologist's perspective by examining the prevalence of dementia diagnoses among patients of a level 4 university hospital dedicated to epilepsy with approximately 5,000 outpatient and 3,000 inpatient visits per year.

It is important to note already at this point that the reviewed cohort is not representative of the broader population of patients who seek medical care for various reasons in neurology, psychiatry, dementia centers, memory clinics, etc. and who may also have epilepsy. It is seizures, i.e., epilepsy, and not another health condition which bring patients to our epilepsy center.

## Methods

**Figure 1:**
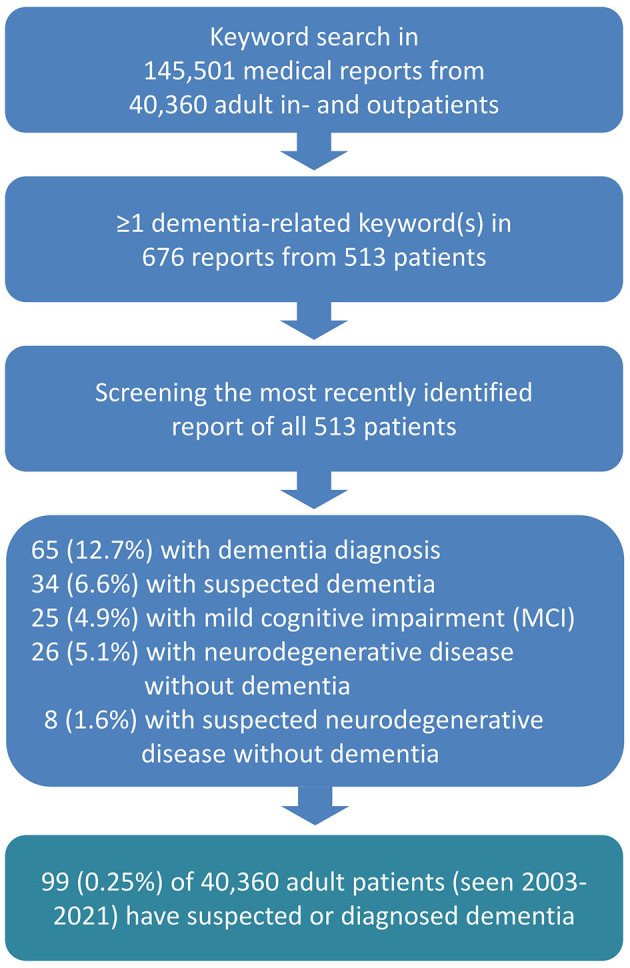
Flowchart.

We conducted a keyword search of a total of 145,501 electronic medical reports of 40,360 adult patients (≥18 years) who visited University Hospital Bonn, Department of Epileptology, from May 2003 to August 2021 (see Figure 1). We used German dementia-related search terms such as ^*^demen^*^, alzheimer^*^, lewy^*^, tauopathie^*^, tau^*^, ^*^amyloid^*^, plaques^*^, neurodegenerat^*^, mci, “mild cognitive”, nootrop^*^, antidemen^*^, and “anti-demen^*^ and did not include conditions such as “encephalopathy” “encephalitis,” “autoimmun^*^,” and “delir^*^,” “status epilepticus,” “NORSE,” or “FIRES,” which can potentially result in dementia even if an explicit dementia diagnosis is not given. We identified 676 reports, and two authors manually screened each of them for type of dementia and comorbid diseases. In cases having multiple reports, we took into account the most recent one, resulting in 513 processed files. We also checked and excluded those letters in which the keywords appeared to be contextually incorrect (e.g., a fear of developing dementia).

Since the reports cover an 18-year period, the old epilepsy nomenclature was used because the new classification system developed by the International League Against Epilepsy (ILAE) was published in 2017 (27). This includes categories such as “no epilepsy” as well as “cryptogenic,” “symptomatic,” and “idiopathic” epilepsy (28). In addition, the temporal relationship between epilepsy and dementia as stated in the medical letters was categorized as dementia before epilepsy, epilepsy before dementia, or unclear. For those adult patients who were 60 years of age or older, we categorized this group of individuals as “older.” Late-onset epilepsy (LOE) was defined as the occurrence of epilepsy in patients who were at least 60 years old at the time of epilepsy onset (29).

The study was approved by the Ethics Committee of the Medical Faculty at the University of Bonn (# 2022 442).

### Statistics

Frequency statistics and cross tabulation with chi-squared test (Chi^2^) and odds ratios (OR) including 95% confidence intervals (CI) were calculated for nominal scaled variables, and ANOVA were conducted for continuous variables. All statistics were performed using R (30) and SPSS (IBM, version 28).

## Results

The medical letters from 513 (1.3%) of 40,360 patients included at least one dementia-related term. In this group, 44% were women and 56% were men. The mean age of the group of 513 patients identified by keyword search was 58.1 ± 18.9 years (range 18–96). Among these patients, clinical diagnoses included symptomatic epilepsy in 270 (52.6%) cases, cryptogenic in 116 (22.6%) cases, idiopathic epilepsy in 73 (14.2%) cases, and no epilepsy in 54 (10.5%) cases. Differential diagnoses in the latter group indicated syncopes or functional non-epileptic seizures. The mean age at epilepsy onset was 45.4 ± 25.4 years (range 0–95), and the average duration was 13.7 ± 17.6 years (range 0–71).

### The prevalence of dementia diagnoses

As for the diagnosis of dementia or a neurodegenerative disease condition, 65 (12.7%) of the 513 patients with medical letters including dementia-related terms had a definitive diagnosis of dementia, and 34 (6.6%) received a suspected dementia diagnosis (Figure 1). The major clinical and demographic characteristics are presented in Table 1, and the exact ICD-11 diagnoses are listed in Table 2.

**Table 1:** Clinical and demographic characteristics of the 99 patients with dementia diagnoses or suspected dementia.

	**Patients with dementia diagnoses or suspected dementia**
**N**	99
**Sex**	
Women	44 (44.4%)
Men	55 (55.6%)
**Age**	
M (SD)	71.4 (15.0)
Range	18–96
**Age at onset of epilepsy**	(*n =* 89)
M (SD)	56.8 (25.0)
Range	3–95
Late-onset epilepsy (≥60 yrs)	50 (56.2%)
**Duration of epilepsy**	(*n =* 89)
M (SD)	14.9 (19.8)
Range	0–71
**Epilepsy diagnosis**	
Structural	64 (64.6%)
Cryptogenic	13 (13.1%)
Idiopathic	13 (13.1%)
No epilepsy	9 (9.1%)
**Dementia diagnosis**	
Yes	65 (65.7%)
Suspected	34 (34.3%)

M, mean; SD, standard deviation.

**Table 2:** Frequencies of different dementia diagnoses (ICD-11).

**Dementia diagnosis**	**Frequency (*n =* 99)**	**ICD-11**
Advanced dementia	2	6D8Z.XS25
Alzheimer's dementia (onset unknown)	7	6D80.Z
Alzheimer's dementia (early onset)	2	6D80.0
Beginning dementia	9	6D8Z.XS5W
Dementia	26	6D8Z
Dementia due to Down Syndrome	1	6D85.9
Fronto-temporal dementia	1	6D83
Fronto-temporal lobar degeneration	2	8A23
Mixed dementia	3	6D80.2
Parkinson's dementia	2	6D85.0
Suspected Lewy-body-dementia DD vascular dementia	1	6D82/6D81
Suspected Alzheimer's dementia (onset unknown)	3	6D80.Z
Suspected Alzheimer's dementia (late onset)	3	6D80.1
Suspected beginning dementia	1	6D8Z.XS5W
Suspected dementia	24	6D8Z
Suspected Lewy-body-dementia	2	6D82
Suspected Lewy-body-dementia DD Creutzfeldt-Jakob disease	1	6D82/6D85.5
Suspected mixed dementia	1	6D80.2
Vascular dementia	7	6D81
Vascular dementia DD fronto-temporal dementia	1	6D81/6D83

By transposing the numbers of diagnosed or suspected dementia cases in the subgroup of 513 patients with medical letters including dementia-related terms (19.3% of 513), we can estimate a prevalence of 0.25% for all 40,360 patients whose files were screened for dementia-related terms.

Table 3 summarizes the reported comorbidities for this group, with the most common ones being arterial hypertension, depression, cerebral infarct, and brain atrophy.

**Table 3:** Comorbidities of patients with dementia diagnoses or suspected dementia.

**Additional diagnosis**	**Frequency (*n =* 99)**
Arterial hypertension	33 (33.3%)
Depression	19 (19.2%)
Cerebral infarct	16 (16.2%)
Brain atrophy	15 (15.2%)
Atrial fibrillation	14 (14.1%)
Polyneuropathy	11 (11.1%)
Traumatic brain injury	11 (11.1%)
Status epilepticus	9 (9.1%)
Coronary artery disease	8 (8.1%)
Delirium	7 (7.1%)
Diabetes mellitus type 2	7 (7.1%)
Microangiopathy	7 (7.1%)
Hippocampal sclerosis	6 (6.1%)
Parkinson's disease	6 (6.1%)
Presbycusis	5 (5.1%)
Hypercholesterolemia	5 (5.1%)
Meningioma	5 (5.1%)
Nicotine addiction	5 (5.1%)
Subarachnoid hemorrhage	5 (5.1%)
Alcohol addiction	4 (4.0%)
Urinary tract infection	4 (4.0%)
Hypothyroidism	4 (4.0%)
Pulmonary embolism	4 (4.0%)
Renal insufficiency	4 (4.0%)
Sleep apnea syndrome	4 (4.0%)
Chronic pain syndrome	4 (4.0%)
Tremor	4 (4.0%)

### Mild cognitive impairment and neurodegenerative diseases without dementia

Among the 513 patients with medical letters including dementia-related terms, 25 (4.9%) patients were diagnosed with mild cognitive impairment (MCI). Additionally, 26 (5.1%) patients were diagnosed with a clear neurodegenerative disease without dementia, while 8 (1.6%) patients were suspected of having a neurodegenerative disease without a definitive dementia diagnosis (Figure 1).

### Potential risk factors for dementia

With regard to age as a risk factor for dementia, in the group of 513 patients who were finally analyzed, the likelihood of having a (suspected) dementia diagnosis increased with advancing age or age at epilepsy onset. Patients with a diagnosis of (suspected) dementia as a group were significantly older (age 71.4 ± 15.0 years vs. 54.9 ± 18.3 years, *F* = 69.5, *p* < 0.001), and the age of their onset of epilepsy was significantly later (age 56.8 ± 25.0 years vs. 41.8 ± 24.5 years, *F* = 25.1, *p* < 0.001) than in patients without (suspected) dementia. The duration of epilepsy was not significantly different between patients with vs. without (suspected) dementia (14.9 ± 19.8 years vs. 13.3 ± 16.9 years, *F* = 0.6, *p* = 0.453).

Using a cutoff age of 60 years, in the younger subgroup (age < 60 years: *n* = 249), 6.8% had a dementia diagnosis or suspected dementia, whereas in the older subgroup (age ≥ 60 years: *n* = 264), there was a significantly higher prevalence of 31.1% [chi^2^ = 48.3(1) *p* < 0.001]. The odds ratio (OR) for the older group compared to the younger group of having diagnosed or suspected dementia was 6.1 (95% CI: 3.5–10.7).

Regarding the age at epilepsy onset, it was observed that in the early-onset subgroup (onset < age 60 years: *n* = 231), 16.9% of the patients received a (suspected) dementia diagnosis. By contrast, the late-onset subgroup (onset ≥ 60 yrs.: *n* = 135) had a significantly higher percentage at 37.0% [chi^2^ = 18.8(1) *p* < 0.001]. For patients with LOE, the odds ratio (OR) of receiving either a dementia diagnosis or a suspected dementia diagnosis was 2.9 (95% CI: 1.7–4.7).

The factors of different types of epilepsy, year of diagnosis, duration of epilepsy, or gender were not found to be associated with a different prevalence of a (suspected) dementia diagnosis (all *p* > 0.1).

### Temporal relationship between epilepsy and dementia

The records of the 99 patients with suspected or diagnosed dementia were reviewed to determine whether the diagnosis of epilepsy or dementia was established first. The dementia diagnosis was made prior to the epilepsy diagnosis for 8.1% of the cases, while in 23.2% of the cases, it was diagnosed after the epilepsy. In the majority of the cases, the time of occurrence was not clearly stated (53.5%) or diagnostics at our center revealed that it was not epilepsy (15.2%).

## Discussion

Given the growing interest among researchers and the public in epilepsy, dementia, and its potentially bidirectional relationship, we sought to investigate the prominence of this issue in a level 4 epilepsy center. This center, and this is of major importance for the understanding of the results of this study, serves as an independent university hospital providing care for in- and out-patients with epilepsy. The pertinent questions raised included the frequency of dementia diagnoses, the level of attention that needs to be directed to this issue, and whether the numbers raise concerns regarding the risk of dementia in patients with epilepsy.

### Prevalence of dementia diagnoses in epilepsy patients seen between 2003 and 2021

Screening was conducted on a total of 145,501 medical letters from all of the 40,360 adult patients who were seen between 2003 and 2021. As a first step, we performed a keyword search of all electronic medical reports to identify dementia-related terms. After multiple reports were excluded, at least one term related to dementia could be identified in the letters of 513 patients. Upon closer examination of these 513 patient files, it was revealed that 99 (19.3%) patients had a confirmed diagnosis of dementia or suspected dementia, while another 59 patients (6.6%) were diagnosed with MCI or (suspected) neurodegenerative conditions without dementia. Thus, no explicit dementia diagnosis was found for most of the 513 patients. Interpolation of these numbers to the total group of 40,360 patients indicates a prevalence of (suspected) dementia of 0.25% seen in a specialized epilepsy center in an 18-year period. As previously mentioned, the investigated patients are not representative of the general epilepsy population, especially not for those experiencing seizures while seeking treatment in neurology, psychiatry, memory, or dementia clinics. Additionally, dementia may be underdiagnosed due to the lack of routine screening for this condition. The diagnostic value of cognitive dementia screenings for identifying MCI or dementia in epilepsy, however, must be questioned. This is because the majority of the patients with chronic epilepsy suffer from cognitive impairments in multiple domains, which, depending on the underlying disease condition, can be substantial and also impact their daily functioning (31–33).

Nevertheless, it is reasonable to assume that highly specialized epilepsy centers tend to receive more patients with severe and difficult-to-treat epilepsies, along with underlying pathologies that increase the risk of developing dementia. In addition, there are those patients seeking advice in specialized epilepsy centers, in whom seizures/epilepsy may be the precursor, i.e., the first sign of a neurodegenerative process.

### Risk factors associated with dementia diagnoses

The likelihood of dementia increases with advancing age. In keeping with existing literature, our findings confirm that an older age (≥60 years) and a later epilepsy onset (≥60 years) significantly change the odds of being diagnosed with (suspected) dementia (22, 23, 25). The odds ratio for (suspected) dementia in persons aged 60 years or older was 6.1. Similarly, for those with LOE, the odds ratio was 2.9, thus highlighting a higher likelihood of (suspected) dementia compared to those with earlier onset epilepsies. The approximately 3-fold risk of dementia in LOE is an interesting and consistently observed finding across multiple studies. However, the prevalence of dementia in our cohort is much lower compared to findings reported in other studies. The most likely explanation for this is a selection bias. For future research, it is of the utmost importance to stratify patients with epilepsy according to the underlying disease conditions and comorbidities that contribute to an increased vulnerability to dementia. A significant distinction exists between patients who primarily experience seizures and seek diagnosis and treatment in specialized epilepsy centers, and those with primarily psychiatric or neurological conditions who are referred to neurology, psychiatry, or dementia clinics where acute seizures or established epilepsies may be perceived as comorbidities. The finding from a recent meta-analysis of observational studies which revealed that patients presenting with LOE also experience a weighted increased risk of 3.88 for a subsequent stroke is thought-provoking. The risk appears similar to that of dementia. The same study also references additional research that provides clinical and radiological evidence to support the premise that LOE is likely associated with underlying cerebrovascular disease (34). Other studies linked antiseizure medication use to a higher risk of vascular events in the general population, and the same risk exists in patients with Alzheimer's disease (15, 35).

Thus, an older age *per se* increases the risk of dementia. Moreover, an older age combined with LOE suggests the presence of acquired brain pathologies, which are more likely degenerative. By contrast, early-onset epilepsies tend to affect brain development (36). Consequently, a recent review demands an exhaustive phenotypic characterization of LOE when the origin is unknown (37).

Remarkably, neither the type of epilepsy nor the duration of epilepsy was related to the diagnosis of (suspected) dementia. The latter finding in particular can be seen as evidence against the notion that suffering from seizures over a prolonged period of time inevitably leads to epileptic dementia. The mean time of dementia ascertainment in the ARIC study was 3.6 years (23). Additionally, comorbidities such as hypertension, depression, cerebral infarct, and brain atrophy have been identified as relevant factors in patients with epilepsy which correspond to the findings reported in other studies (25).

Understanding the timely and potentially causal relationship between epilepsy and the diagnosis of dementia is crucial. According to the medical reports epilepsy tends to precede dementia more frequently than vice versa. This is consistent with the observations made in the ARIC study (23). In patients with LOE, seizures can be perceived as an early biomarker of subsequent neurological diseases including dementia. Etiological considerations and bidirectional contingencies are worth to be addressed with population- and registry-based studies.

### Limitations

This study has several limitations. First, the diagnosis of dementia according to medical letters might underestimate the real prevalence as an epilepsy center does not aim at dementia as such but at epilepsy-related issues. Therefore, although dementia may be present, it may not be referenced in medical letters. Second, the patient collection is not representative of the general epilepsy population. Additionally, we did not have the resources to inspect all 40,360 patient files for a closer description of the patient cohort. As for the selection bias, it is reasonable to assume that patients with more severe and pharmacoresistant epilepsies who are seen in a highly specialized university center solely dedicated to epilepsy may have a higher likelihood of developing dementia than those patients who are more easily treatable and seek care elsewhere. Furthermore, the data do not allow for the calculation of a point prevalence. Thus, a comparison between the prevalence of dementia in our study against the general population was not possible.

## Conclusion

Considering the selection bias, the absence of a non-epilepsy control group, and the potential underdiagnosis of dementia in an epilepsy center, the main finding of this analysis is that from the epileptological perspective, dementia appears to be a relatively rare phenomenon. It is less likely to be solely attributed to experiencing seizures or having chronic epilepsy, but rather to seizures being a symptom of critical underlying pathologies and comorbidities that connect epilepsy to dementia (11). Physicians need to be aware of dementia, especially in elderly patients and in those with late-onset epilepsies. Future research should focus on explicitly screening for dementia in epilepsy, while also stratifying patients according to their underlying pathologies and comorbidities.

## Data availability statement

The raw data supporting the conclusions of this article will be made available by the authors, without undue reservation.

## Ethics statement

The studies involving humans were approved by Ethics Committee of the Medical Faculty at the University of Bonn. The studies were conducted in accordance with the local legislation and institutional requirements. Written informed consent for participation was not required from the participants or the participants' legal guardians/next of kin because it is a retrospective analysis of anonymized clinical data.

## Author contributions

CH and J-AW conceptualized the research topic. CH, TL, and J-AW wrote the manuscript. VW and TL conducted the data extraction and statistical analyses. All authors contributed to the article and approved the submitted version.

## References

[1] Helmstaedter C Witt JA. Clinical neuropsychology in epilepsy: theoretical and practical issues. Handb Clin Neurol. (2012) 107:437–59. 10.1016/B978-0-444-52898-8.00036-7 22938988

[2] Helmstaedter C Witt JA. Epilepsy and cognition - A bidirectional relationship? Seizure. (2017) 49:83–9. 10.1016/j.seizure.2017.02.017 28284559

[3] Elger CE Helmstaedter C Kurthen M. Chronic epilepsy and cognition. Lancet Neurol. (2004) 3:663–72. 10.1016/S1474-4422(04)00906-8 15488459

[4] Kaaden S Helmstaedter C. Age at onset of epilepsy as a determinant of intellectual impairment in temporal lobe epilepsy. Epilepsy Behav. (2009) 15:213–7. 10.1016/j.yebeh.2009.03.027 19318136

[5] Breuer LE Boon P Bergmans JW Mess WH Besseling RM de Louw A. Cognitive deterioration in adult epilepsy: Does accelerated cognitive ageing exist? Neurosci Biobehav Rev. (2016) 64:1–11. 10.1016/j.neubiorev.2016.02.004 26900650

[6] Helmstaedter C Elger CE. The phantom of progressive dementia in epilepsy. Lancet. (1999) 354:2133–4. 10.1016/S0140-6736(99)03542-4 10609823

[7] Kraepelin E. Psychiatrie. Ein Lehrbuch für Studierende und Aerzte Sechste Vollständig Überarbeitete Auflage. Leipzig: Verlag von Johann Ambrosius Barth (1899).

[8] Bumke O. Die Diagnose der Geisteskrankheiten. Wiesbaden: Verlag von FJ Bergmann. (1919).

[9] Beghi E Giussani G Costa C DiFrancesco JC Dhakar M Leppik I. The epidemiology of epilepsy in older adults: a narrative review by the ILAE task force on epilepsy in the elderly. Epilepsia. (2023) 64:586–601. 10.1111/epi.17494 36625133

[10] Piccenna L O'Dwyer R Leppik I Beghi E Giussani G Costa C. Management of epilepsy in older adults: a critical review by the ILAE task force on epilepsy in the elderly. Epilepsia. (2023) 64:567–85. 10.1111/epi.17426 36266921

[11] Sen A Capelli V Husain M. Cognition and dementia in older patients with epilepsy. Brain. (2018) 141:1592–608. 10.1093/brain/awy022 29506031 PMC5972564

[12] Thompson PJ Duncan JS. Cognitive decline in severe intractable epilepsy. Epilepsia. (2005) 46:1780–7. 10.1111/j.1528-1167.2005.00279.x 16302858

[13] Dodrill CB. Progressive cognitive decline in adolescents and adults with epilepsy. Prog Brain Res. (2002) 135:399–407. 10.1016/S0079-6123(02)35037-4 12143358

[14] Dodrill CB Wilensky AJ. Intellectual impairment as an outcome of status epilepticus. Neurology. (1990) 40:23–7. 2185437

[15] Taipale H Gomm W Broich K Maier W Tolppanen AM Tanskanen A. Use of antiepileptic drugs and dementia risk-an analysis of Finnish health register and German health insurance data. J Am Geriatr Soc. (2018) 66:1123–9. 10.1111/jgs.15358 29566430

[16] Mo L Ding X Tan C Liu X Wei X Wang H. Association of cerebrospinal fluid zinc-alpha2-glycoprotein and tau protein with temporal lobe epilepsy and related white matter impairment. Neuroreport. (2019) 30:586–91. 10.1097/WNR.0000000000001252 30964766

[17] Keret O Hoang TD Xia F Rosen HJ Yaffe K. Association of late-onset unprovoked seizures of unknown etiology with the risk of developing dementia in older veterans. JAMA Neurol. (2020) 77:710–5. 10.1001/jamaneurol.2020.0187 32150220 PMC7063560

[18] Lam AD Sarkis RA Pellerin KR Jing J Dworetzky BA Hoch DB. Association of epileptiform abnormalities and seizures in Alzheimer disease. Neurology. (2020) 95:e2259–70. 10.1212/WNL.0000000000010612 32764101 PMC7713786

[19] Subota A Pham T Jette N Sauro K Lorenzetti D Holroyd-Leduc J. The association between dementia and epilepsy: a systematic review and meta-analysis. Epilepsia. (2017) 58:962–72. 10.1111/epi.13744 28397967

[20] Huang L Fu C Li J Peng S. Late-onset epilepsy and the risk of dementia: a systematic review and meta-analysis. Aging Clin Exp Res. (2022) 34:1771–9. 10.1007/s40520-022-02118-8 35428922

[21] Tang T Zhang R Pan X. Meta-analysis of the risk of dementia in elderly patients with late-onset epilepsy. Clin Neurol Neurosurg. (2022) 223:107499. 10.1016/j.clineuro.2022.107499 36327952

[22] Johnson EL Krauss GL Walker KA Brandt J Kucharska-Newton A Mosley TH. Late-onset epilepsy and 25-year cognitive change: the atherosclerosis risk in communities (ARIC) study. Epilepsia. (2020) 61:1764–73. 10.1111/epi.16616 32710450 PMC7718433

[23] Johnson EL Krauss GL Kucharska-Newton A Albert MS Brandt J Walker KA. Dementia in late-onset epilepsy: the atherosclerosis risk in communities study. Neurology. (2020) 95:e3248–e56. 10.1212/WNL.0000000000011080 33097597 PMC7836657

[24] Stefanidou M Beiser AS Himali JJ Peng TJ Devinsky O Seshadri S. Bi-directional association between epilepsy and dementia: the Framingham heart study. Neurology. (2020) 95:e3241–e7. 10.1212/WNL.0000000000011077 33097599 PMC7836659

[25] Tsai ZR Zhang HW Tseng CH Peng HC Kok VC Li GP. Late-onset epilepsy and subsequent increased risk of dementia. Aging. (2021) 13:3573–87. 10.18632/aging.202299 33429365 PMC7906153

[26] Ophir K Ran B Felix B Amir G. Ten year cumulative incidence of dementia after late onset epilepsy of unknown etiology. J Clin Neurosci. (2021) 86:247–51. 10.1016/j.jocn.2021.01.030 33775336

[27] Scheffer IE Berkovic S Capovilla G Connolly MB French J Guilhoto L. ILAE classification of the epilepsies: position paper of the ILAE commission for classification and terminology. Epilepsia. (2017) 58:512–21. 10.1111/epi.13709 28276062 PMC5386840

[28] Commission on Classification and Terminology of the International League Against Epilepsy. Proposal for revised classification of epilepsies and epileptic syndromes. Epilepsia. (1989) 30:389–99. 10.1111/j.1528-1157.1989.tb05316.x 2502382

[29] Witt JA Werhahn KJ Kramer G Ruckes C Trinka E Helmstaedter C. Cognitive-behavioral screening in elderly patients with new-onset epilepsy before treatment. Acta Neurol Scand. (2014) 130:172–7. 10.1111/ane.12260 24796793

[30] R Core Team. The R Project for Statistical Computing. (2021). Available online at: https://www.R-project.org

[31] Witt JA Meschede C Helmstaedter C. Hazardous employment of invalid measures for cognitive outcome assessment: you only see what your test can show you. Epilepsy Behav. (2021) 117:107865. 10.1016/j.yebeh.2021.107865 33662843

[32] Kaestner E Reyes A Chen A Rao J Macari AC Choi JY. Atrophy and cognitive profiles in older adults with temporal lobe epilepsy are similar to mild cognitive impairment. Brain. (2021) 144:236–50. 10.1093/brain/awaa397 33279986 PMC7880670

[33] Norman M Wilson SJ Baxendale S Barr W Block C Busch RM. Addressing neuropsychological diagnostics in adults with epilepsy: introducing the international classification of cognitive disorders in epilepsy: the IC CODE initiative. Epilepsia Open. (2021) 6:266–75. 10.1002/epi4.12478 34033259 PMC8166800

[34] Wall J Knight J Emsley HCA. Late-onset epilepsy predicts stroke: systematic review and meta-analysis. Epilepsy Behav. (2021) 115:107634. 10.1016/j.yebeh.2020.107634 33334717

[35] Sarycheva T Lavikainen P Taipale H Tiihonen J Tanskanen A Hartikainen S. Antiepileptic drug use and the risk of stroke among community-dwelling people with Alzheimer disease: a matched cohort study. J Am Heart Assoc. (2018) 7:e009742. 10.1161/JAHA.118.009742 30371186 PMC6222965

[36] Helmstaedter C Elger CE. Chronic temporal lobe epilepsy: a neurodevelopmental or progressively dementing disease? Brain. (2009) 132:2822–30. 10.1093/brain/awp182 19635728

[37] DiFrancesco JC Labate A Romoli M Chipi E Salvadori N Galimberti CA. Clinical and instrumental characterization of patients with late-onset epilepsy. Front Neurol. (2022) 13:851897. 10.3389/fneur.2022.851897 35359649 PMC8963711

